# Fourth-generation gene editors: Integration-based genome engineering

**DOI:** 10.1016/j.omta.2026.201753

**Published:** 2026-05-07

**Authors:** Aidan E. Kincaid, Alex W. Hewitt, Rajendra KC

**Affiliations:** 1 Alphinia Bio, Lenah Valley, Hobart, TAS 7008, Australia; 2 Menzies Institute for Medical Research, University of Tasmania, Hobart, TAS 7000, Australia; 3 School of Medicine, University of Tasmania, Hobart, TAS 7000, Australia; 4 Centre for Eye Research Australia, University of Melbourne, Melbourne, VIC 3002, Australia

**Keywords:** gene editors, genome engineering, gene therapy, therapeutic gene integration, site-specific integration

## Abstract

Gene editing has rapidly progressed from early methods into highly precise tools with immense therapeutic promise. While initial CRISPR-Cas9 systems revolutionized the field, the fact that they rely on double-strand breaks can lead to genomic instability and off-target effects, which calls for innovation. This review covers the advancements in fourth-generation gene editing technologies. Specifically, it focuses on diverse integrase systems that enable precise DSB-free DNA insertion. First, we explore site-specific recombinases like tyrosine recombinases (Cre, FLP), large serine recombinases (PhiC31, Bxb1), and bridge recombinases (IS622) that offer efficient DNA rearrangements. Next, we introduce DNA transposases, which cover a versatile cut and paste systems (*piggyBac*, *Sleeping Beauty*), as well as the CRISPR-associated transposons (CASTs, type I-F, type V-K), which achieve RNA-guided, DSB-free DNA insertion with high efficiency and directionality. This review also highlights viral integrases (HIV, MLV), known for their stable gene delivery, and mobile group II introns (targetrons) and R2 retrotransposons for their site-specific integration and all-RNA delivery strengths. Even though there have been significant advances in efficiency, specificity, and delivery strategies, challenges with these powerful tools remain. Continued refinement of these integrase systems will facilitate a new generation in gene-based approaches for the definitive correction of variants of inherited diseases.

## Graphical abstract

**Figure:**
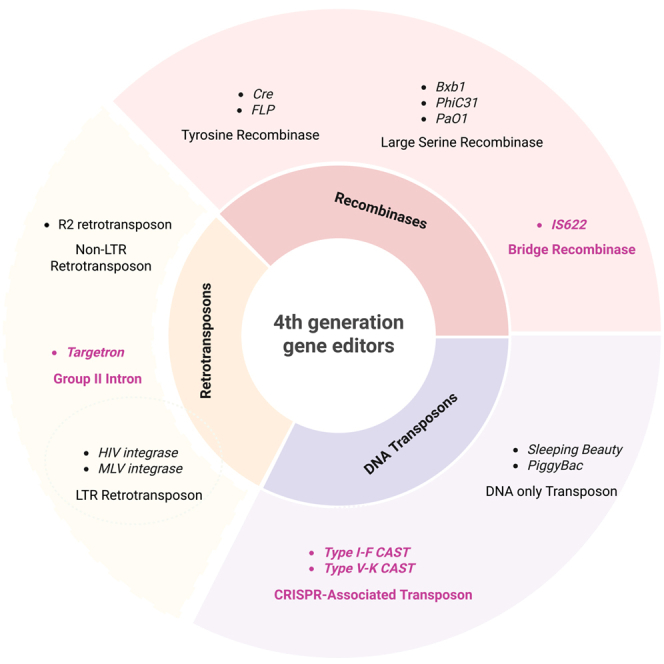

## Teaser

KC and colleagues reviewed emerging integrase-based gene editors capable of inserting large DNA sequences in the genome, highlighting their efficiency, cargo capacity, programmability and delivery strategies for potential clinical application.

## Introduction

**Figure 1:**
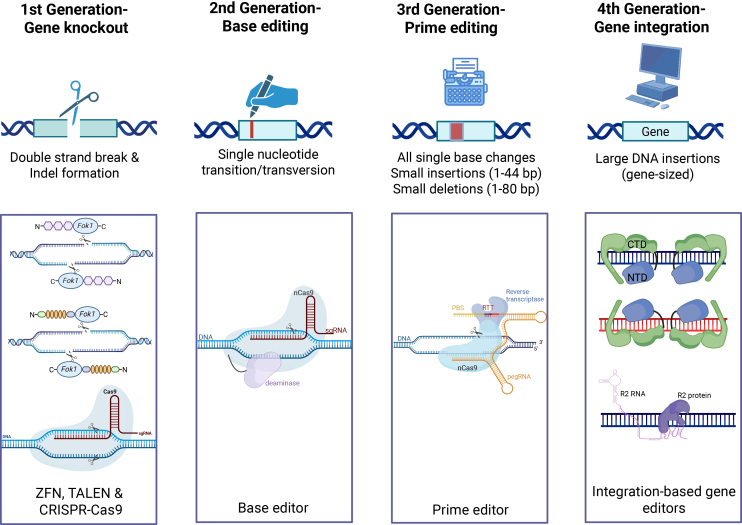
Overview of four generations of gene editing tools First-generation gene editing tools like zinc finger nucleases (ZFNs), transcription activator-like effector nucleases (TALENs) and CRISPR-Cas9 are like molecular scissors that cut both strands of DNA to create knockouts through error-prone repair pathway. Second-generation tools, base editors, are like a DNA pencil that can precisely change a single nucleotide (transition or transversion) without creating a double-strand break. Third-generation tools, prime editors, are like DNA typewriters that can perform small DNA conversions, including all single base changes, small insertions, and deletions. Fourth-generation gene editing technology will include integration-based tools that are capable of inserting large gene-sized DNA at a specific genomic locus. This figure was created in BioRender.

Genome engineering has had remarkable progress in the last two decades. So far, this technology has gone through four main generations of development (Figure 1), each overcoming key limitations of its predecessors. Although we use “generations” as a convenient framework, it is not meant to imply that all fourth-generation technologies were discovered after CRISPR. Many of the large gene integration strategies covered in this paper, including site-specific recombinases (SSRs), transposases, and retrotransposons, were described and experimentally studied long before the mechanistic characterization of CRISPR-Cas9 as a programmable RNA-guided nuclease in 2012.^1^ Early studies demonstrated that several of these integration systems could function in mammalian cells and were even proposed as potential gene therapy tools, including transposon-based platforms such as *Sleeping Beauty*^2^ and *piggyBac,*^2^^,^^3^ as well as site-specific recombinase systems such as Cre and Bxb1 recombinases.^4^^,^^5^^,^^6^ However, recent advances in molecular engineering, protein design, and targeting strategies have renewed interest in these systems and enabled their development as programmable genome engineering tools. In this review, we use the phrase “fourth-generation” to describe the renewed development of integration-based genome editing systems that have the potential to insert gene-sized DNA into a genome without double-strand breaks (DSBs). This reflects that there has been a shift in editors that make small DNA changes to platforms designed to insert larger therapeutic genes into the genome.

The first-generation of gene editing tools included the meganuclease, zinc finger nucleases (ZFNs), and transcription activator-like effector nucleases (TALENs) and clustered regularly interspaced short palindromic repeats (CRISPR)-Cas9 systems.^7^^,^^8^^,^^9^^,^^10^^,^^11^ ZFNs and TALENs rely on protein-DNA recognition, while CRISPR-Cas9 systems use guide RNA to direct their nucleases to user-specified DNA sequences. At the target site, nucleases cut both strands of DNA and induce DSBs, which are then repaired by two major host repair mechanisms: non-homologous end joining (NHEJ) or homology-directed repair (HDR) pathways. The NHEJ pathway is predominant and error-prone, often resulting in small insertions or deletions (indels), thereby disrupting the gene and creating knockouts.^12^ The HDR pathway, on the other hand, allows more precise gene insertions or corrections when a homologous donor template is supplied.^13^ However, HDR-mediated gene insertion is highly inefficient, cell-cycle dependent, and essentially inactive in non-dividing cells, making them unsuitable for precise gene insertions in most therapeutic contexts.

After that came the second-generation base editors that allowed precise, single base conversions without creating a DSB.^14^^,^^15^ Classical base editors cytosine and adenine base editors were limited to transition substitutions (C to T or A to G);1^14^^,^^15^ however, more recent glycosylase base editors and adenine transversion editors have enabled C to A or G and A to C transversions, respectively.^16^^,^^17^ However, a known limitation of these base editing technologies is the occurrence of bystander edits due to the promiscuous activity of fused deaminases.^18^ Such bystander edits may introduce missense mutations, posing challenges for therapeutic applications. Furthermore, base editors exhibit substantial gRNA-dependent off-target activity at unintended genomic loci.^19^ Together, these bystander and off-target effects present significant limitations for the clinical translation of base editing technologies.

The third-generation prime editors further expanded the scope of precise gene editing by using an engineered reverse transcriptase and prime editing guide RNA (pegRNA) to carry out small DNA conversions, which includes all possible single base conversions, small insertions (up to 44 bp) and small deletions (up to 80 bp).^20^ Both base editors and prime editors do not rely on error-prone DSBs and inefficient HDR pathways, and are functional in both dividing and non-dividing cells. However, these technologies remain limited in the size of DNA they can edit, restricting their use for therapeutic gene-sized insertions. Additionally, both base editing and prime editing have recently been reported to generate DSB and genotoxic byproducts, including large deletions and translocations, albeit at a lower frequency than Cas9, limiting their application in clinical settings.^21^^,^^22^

Recent progress has further advanced the capacity of prime editing for large-scale genomic modifications. Prime assembly uses twin prime editing to generate paired 3′ DNA flaps on both the genome and donor DNA, which join together to allow for large scarless DNA insertions.^23^^,^^24^ Notably, prime assembly activity was found similar in both dividing and non-dividing cells and was capable of inserting cargos of up to 11 kb at therapeutically safe loci in human cells. Prime assembly thus represents a scalable extension of prime editing that bridges the gap between small precise edits and large-scale targeted insertions.

Beyond reverse transcriptase-based systems, emerging polymerase-based genome writing platforms offer a complementary approach to precise DNA editing. DNA polymerase editing (DPE) leverages the phi29 DNA polymerase to achieve editing efficiencies of up to 60% in human cells with support for insertions exceeding 100 nucleotides.^25^ Click editing, on the other hand couples HUH endonuclease-mediated template recruitment with a DNA-dependent polymerase to install precise edits with minimal indels across diverse human cell types.^26^ Unlike reverse transcriptase-based editors, both systems use DNA-dependent DNA polymerases in place of reverse transcriptases, which offer higher fidelity and processivity for genome writing. These platforms demonstrate that the search for optimal genome writing enzymes extends beyond reverse transcriptases and may inform the continued evolution of integration-based editing strategies.

Several genome editing technologies from the first three generations are now entering clinical translation. For example, CRISPR-Cas9-based therapies have shown success *in vivo* genome editing in human patients. Specifically, the treatment of transthyretin (TTR) amyloidosis was made with lipid nanoparticle (LNP) delivery of CRISPR-Cas9 components, through targeted knockout of TTR.^27^^,^^28^ In parallel with this, *ex vivo* CRISPR editing approaches are being used to treat hematological disorders like sickle cell disease and β-thalassemia.^29^ This therapy, Casgevy, is the world’s first FDA-approved CRISPR-Cas9 gene editing therapy.^29^^,^^30^ Base editing technologies have also taken significant steps toward clinical testing, with early therapeutic programs targeting hematologic and genetic diseases and currently are in phase 1/2 clinical trials. Key trials include VERVE-101 for familial hypercholesterolemia,^31^ BE-CAR7 for T cell acute lymphoblastic leukemia,^32^ BEAM-302 for alpha-1 antitrypsin deficiency (NCT06389877).

Finally, the field has entered the era of the fourth-generation of gene editors. These include recombinases and integrases, which unlike earlier generations can directly insert large DNA payloads into defined genomic loci without reliance on HDR or DSB repair. The ability to install larger genomic edits is essential for enabling mutation-agnostic therapeutic strategies. Analysis of the ClinVar database revealed 75,122 known pathogenic human genetic variants, distributed across diverse mutation types including point mutations, insertions, deletions, and duplications.^20^ While conventional base editing and prime editing can address individual small variants, many disease genes harbor hundreds of distinct pathogenic alleles scattered across multiple exons. Correcting each variant individually would require bespoke reagent design and validation, limiting clinical scalability. Mutation-agnostic approaches, such as full exon replacement or transgene integration, offer a single therapeutic strategy applicable to all patients carrying mutations within a given gene or exon, irrespective of the specific variant. Such strategies require the installation of kilobase-scale edits that exceed the capacity of current base editors and standard prime editors. This new generation offers the potential for safe, efficient, and mutation-agnostic gene replacement therapies. However, current integration-based platforms still lack comprehensive efficiency, specificity, and long-term safety profiling. This is particularly evident in clinically relevant primary cell types and *in vivo* settings, which underscore the need for continued engineering and preclinical evaluation before they can be used in a widespread therapeutic setting.

**Figure 2:**
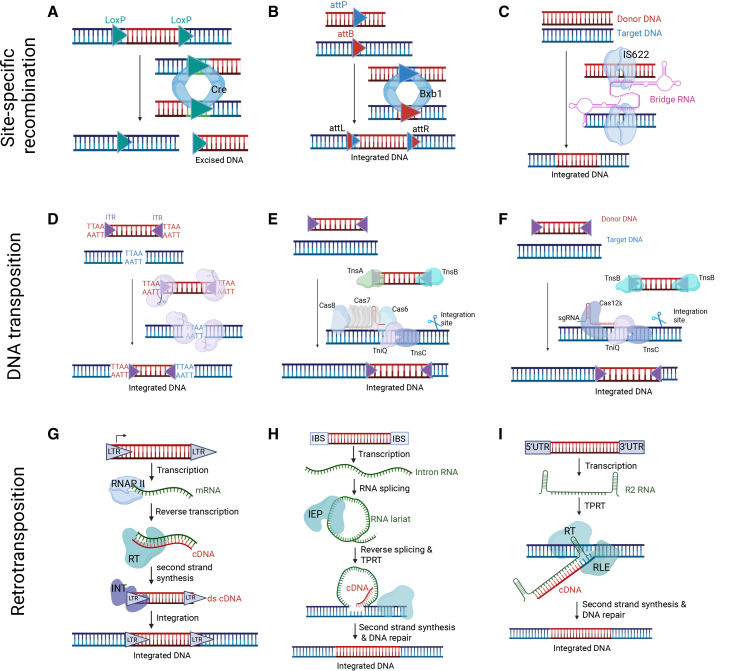
Schematic representation of the mechanism for DNA integration across three major classes of integration systems; recombination, DNA transposition, and retro-transposition (A–C) Represent the mechanism of site-specific recombinases. (A) Diagram illustrating the mechanism of tyrosine recombinase representative Cre that recognizes specific *loxP* sequence to catalyze precise large DNA excision. (B) Large serine recombinase representative Bxb1 recognizes specific attP sequence in the donor DNA and attB in the target DNA to catalyze large DNA insertions. (C) Bridge recombinase representative IS622 mediates large DNA insertions via its cognate bridge RNA that recognizes and base-pairs with specific sequences in the donor and target DNA. (D–F) Represent the mechanism of DNA transposases for cut and paste transposition. (D) DNA-only transposase representative piggyBac recognizes inverted terminal repeats (ITRs) and cuts the cargo from the donor DNA and inserts into a specific TTAA site in the target DNA. (E) Type I-F CAST (CRISPR-associated transposon) system uses CRISPR-Cas proteins (Cas6, Cas7, and Cas8), TniQ, and transposases (Tns A, B, and C) to direct the insertion of donor DNA (recognized by TnsA and B) into a specific target DNA site guided by a small guide RNA (sgRNA). (F) Type V-K CAST system uses Cas12 k, TniQ, and transposases (Tns B and C) to direct the insertion of donor DNA (recognized by TnsB) into a specific target DNA site guided by a sgRNA. (G–I) Represent the mechanism of Retrotransposases for copy and paste transposition. (G) LTR (long terminal repeat) retrotransposon system involves transcription of LTR-flanked cargo followed by reverse transcription of the mRNA intermediate to create complementary DNA (cDNA), which is then integrated into target DNA catalyzed by an associated integrase and host factors. (H) Group II introns representative targetrons utilizes intron encoded protein (IEP) and intron RNA to catalyze RNA splicing to form an RNA lariat, followed by reverse splicing and target-primed reverse transcriptase (TPRT) for inserting cargo into a specific target DNA site determined by the intron binding sequence (IBS) in the intron RNA. (I) Non-LTR retrotransposon representative R2 retrotransposon system mediates site-specific integration into 28 S rDNA locus via TPRT mechanism mediated by R2 RNA and R2 protein. The R2 protein has DNA binding domains, a reverse transcriptase (RT) and a restriction-like endonuclease (RLE) domain. Target site specificity is determined by DNA binding domains of the R2 protein and R2 RNA homology arms. This figure was created in BioRender.

**Figure 3:**
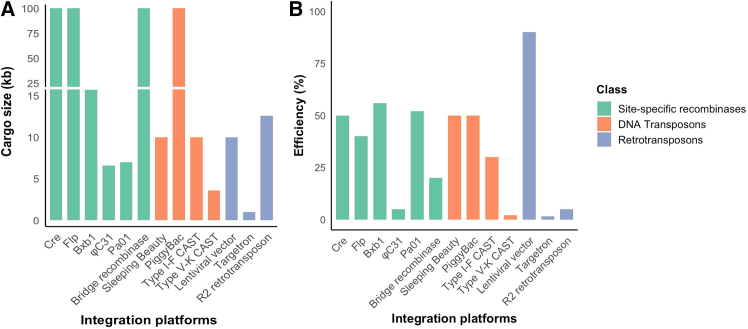
Comparison of maximum reported cargo capacity and integration efficiencies across different fourth-generation gene editors (A) Represents the maximum cargo size in kilobase that was published in studies or source datasets where the integration system was tested. (B) Demonstrates the highest reported integration efficiencies (%) for mammalian systems (HEK293T, embryonic stem cells, or primary fibroblasts). There are no error bars because the values are literature-reported maximums and not averaged over other experiments. (A and B) Compares the performance of the platforms in delivering capacity and genomic integration efficiency. Integration efficiency is highly context-dependent and varies with genomic locus, chromatin state, epigenetic features, cell type, and experimental design. The values presented here represent the best-case scenarios under specific experimental conditions and should not be directly compared across platforms without considering these variables. While some platforms support very large cargo sizes, many therapeutically relevant genes fall within the ∼1–3 kb range, meaning that even moderate-capacity systems could be highly valuable for clinical applications. This figure was created in R using ggplot2.

This review will provide an overview of the current generation of gene editors for large DNA integration, presented in three major classes based on their core mechanism and catalytic enzyme, namely SSRs, DNA transposons, and retrotransposons (Figure 2). We have also included recent engineering advances, and their therapeutic potential for treating genetic disorders. The major features of these editors, including cargo capacity, editing efficiencies, targeting modes, ease of programmability, and delivery strategies are summarized in Figure 3 and Table 1.

**Table 1:** Comparative features of 4th-generation genome editors for large DNA integration

Class	Mechanism	Platform	Targeting mode	Ease of programmability	Reported delivery strategy	Reference
Site-specific recombinase	DNA to DNA recombination	Cre	site-specific; recognizes *loxP* sites	hard (protein engineering)	plasmid DNA or viral vectors	39
		FLP	site-specific; recognize FRT site	hard (protein engineering)	plasmid DNA or viral vectors	40
		Bxb1	site-specific; attB and attP sites	easy for Cas9 fusion or medium for ZF/TALE fusions or hard for unfused original protein	plasmid DNA (integrase + donor ± PE)	46
		φC31	site-specific; attB and attP sites	easy for Cas9 fusion or medium for ZF/TALE fusions or hard for unfused original protein	plasmid DNA (integrase + donor ± PE)	46
		Pa01	site-specific; attB and attP sites	easy for Cas9 fusion or medium for ZF/TALE fusions or hard for unfused original protein	plasmid DNA (integrase + donor)	34,43
	RNA-guided DNA to DNA recombination	bridge recombinase IS622	site-specific; RNA-guided via bridge RNA	easy (RNA-guided)	plasmid DNA or viral vectors and potential all-RNA delivery	49
DNA transposase	cut and paste integration	*Sleeping Beauty* (SB100×)	semi-random; inserts at TA sites	hard (protein engineering)	plasmid transposon + transposase	51
		piggyBac	semi-random; inserts at TTAA sites	easy for Cas9 fusion or Medium for ZF/TALE fusions or Hard for unfused original protein	plasmid transposon + transposase	54,56
		type I-F CAST	site-specific; RNA-guided via Cascade-crRNA	easy (RNA-guided)	plasmid DNA or DNA-based viral vectors	66
		type V-K CAST	site-specific; RNA-guided via Cas12 k-sgRNA	easy (RNA-guided)	plasmid (Cas12 k system + donor)	68
Retrotransposase	copy and paste integration	retroviral integrase	semi-random; inserts at transcriptionally active sites	hard (protein engineering of integrase)	–	–
		Targetron	site-specific; intron RNA exon binding sequence base pairing with the target sequence	easy (intron RNA by base pairing rule)	plasmid (RNP expression) + Mg^2+^	79
		R2 retrotransposons	site-specific; 28 S rDNA via DNA binding domains in the R2 protein	easy for Cas9 fusion or medium for ZF fusion or hard for unfused original protein	All-RNA based delivery	89

Re-programmability of integration-based platforms is classified into 3 tiers based on complexity of retargeting. Platforms with “easy” re-programmability are those in which target-site specificity is encoded by a short RNA molecule, like guide RNA and bridge RNA, and does not require any modification to the effector protein. “Medium” re-programmability applies to systems that use a single modular DNA-binding domain, such as zinc finger or TALE arrays fused to recombinases or transposases. These follow well-characterized recognition codes but require protein engineering for retargeting. “Hard” re-programmability applies to systems with multi-component or non-modular DNA-binding interfaces, such as integrases, which recognize fixed attachment sites through complex protein-DNA contacts. Retargeting these systems requires extensive-directed evolution and has been achieved only in limited cases. The “reference” column indicates representative studies demonstrating each system, including validation in mammalian or human cell contexts where applicable. The “delivery” column shows the different delivery methods that were used in the referenced studies (e.g., plasmid transfection, mRNA delivery, viral vectors, or RNP delivery). There was not a standardized delivery approach for the systems.

### SSRs

SSRs are enzymes that catalyze precise DNA rearrangements through a highly specific process illustrated in Figures 2A–2C. First, SSRs will recognize DNA sequences ranging from 30 to 50 base pairs. Once they recognize the DNA sequence, the SSRs will make precise cuts to either excise, integrate, or invert DNA segments through a strand exchange mechanism that proceeds via covalent recombinase-DNA intermediates, without generating free DNA ends, making them attractive for safe genomic engineering.^33^^,^^34^ Moreover, what makes these recombinases interesting is that, unlike homologous recombination, SSRs do not require extensive sequence homology. This makes them much more efficient and a precise tool for genome engineering. In today’s research, these recombinases are used in gene manipulation, gene circuit assemblies, and transgenic organisms. There are 3 major families of SSRs: tyrosine recombinases, large serine recombinases (LSRs), and bridge recombinases, which differ in their catalytic mechanisms and biological origins.

### Tyrosine recombinases

Tyrosine recombinases such as Cre and FLP are widely used for conditional knockout models and cassette exchange.^35^ They recombine between two identical 34-base-pair (bp) *loxP* or *FRT* sites to excise (Figure 2A), integrate, or invert DNA in a highly predictable manner.^36^ Mechanistically, four recombinase molecules assemble with two recognition sites in an antiparallel orientation. The catalytic tyrosine residue acts as a nucleophile, attacking the scissile phosphodiester bond to form a covalent 3′-phosphotyrosine intermediate and releases a free 5′-hydroxyl end.^37^ Strand exchange between the two sites generates a Holliday junction intermediate, which is resolved by cleavage and exchange of the second pair of strands to yield recombinant products.^38^ Importantly, this reaction proceeds without requiring ATP or additional host factors.

Cre recombination is highly efficient, and can even reach up to 70% in mammalian cells; however, it lacks directional control needed to stabilize the integration product.^34^^,^^39^ FLP also follows bidirectional recombination but at a lower efficiency compared to Cre. Research has allowed for engineered variants like FLPe (4-fold increase) and codon-optimized FLPo that have significantly improved their efficiency almost to the levels of Cre.^34^^,^^40^

Despite their high efficiency and predictability, tyrosine recombinases have two fundamental limitations for therapeutic gene integration. First, they require pre-installation of recognition sites (*loxP* or FRT) at the target locus, as their specificity is determined entirely by protein-DNA contacts that cannot be readily reprogrammed to recognize novel genomic sequences. Second, the reversibility of the recombination reaction favors excision over integration, making these systems better suited for gene knockout rather than stable transgene integration applications genome engineering.^41^

### LSRs

LSRs are bacteriophage-derived enzymes that use unidirectional DNA integration between two non-identical recognition sites.^42^ The big integrases from this family involve classical phage-derived LSRs, PhiC31, and Bxb1, and a newly described Pa01 identified from metagenomic mining.^6^^,^^43^^,^^44^ Unlike tyrosine recombinase, they mediate unidirectional recombination between specific attachment sites attB and attP through the creation of hybrid attL and attR sites that are no longer substrates for recombination (Figure 2B).^45^ This unidirectionality is important as it makes them a more stable and irreversible form of gene integration and hence, an attractive tool for therapeutic integration.

Research on PhiC31 shows that integration efficiencies can range from 0.7% to 3.0% in cell lines such as HeLa, HEK293, and NIH3T3 with a pre-inserted PhiC31 attP site, though evolved variants can reach approximately 18%.^46^ The same study reported integration efficiencies up to 60% for evolved Bxb1 variants in clonal HEK293 cell line containing a pre-inserted Bxb1 attB site. Importantly, evolved Bxb1 variants retained robust activity across diverse human cell types, including HeLa, U2OS, K562 lines and even primary human fibroblasts, demonstrating its suitability for translational application. Compared to both evolved PhiC31 and Bxb1, Pa01 consistently has shown higher integration efficiency up to 75% for cargos >7 kb.^43^ Evolving Pa01 could push integration efficiencies even higher and therefore could establish it as a gold-standard integrase, surpassing even Bxb1 for translational applications.

LSRs have shown promising results in mammalian cells and the capacity to integrate large DNA payloads up to 15 kb, making them important tools in the realm of synthetic biology. This process allows for stable and permanent gene insertion. This trait of LSRs makes them powerful tools for long-term modifications in systems.

### Bridge recombinases

Unlike tyrosine recombinases and LSRs that rely on DNA-based landing pads, bridge recombinase represents an RNA-guided recombinase platform consisting of a non-coding bridge RNA and a recombinase enzyme to achieve precise genomic rearrangements (Figure 2C).^47^ The bridge RNA contains two independent programmable internal loops, a target-binding loop (TBL) and a donor-binding loop (DBL), that can bp with the target DNA and the donor DNA site, respectively.^47^ Each loop contains variable guide segments that bp with the top and bottom strands of their respective DNA targets, converging at a conserved core dinucleotide essential for recombination.^48^ This bispecific architecture is unique among RNA-guided systems, as a single RNA molecule simultaneously specifies both the genomic target and the donor sequence. Importantly, the TBL and DBL can be independently reprogrammed without modification to the recombinase protein, conferring RNA-only re-targetability comparable to CRISPR-guide RNAs, and making the system inherently compatible with all-RNA LNP delivery.

Upon binding, the recombinase is recruited to the complex, bringing the two sites into proximity and catalyzing the recombination reaction through top-strand cleavage and exchange, Holliday junction formation, and bottom-strand cleavage and exchange.^48^ Initially, they were reported as programmable tools for modifying prokaryotic genomes. Very recently, Perry et al. discovered IS622, a bridge recombinase ortholog active in human cells.^49^ Through rational engineering of the recombinase and bridge RNAs, the efficiency and specificity of insertion was enhanced to as high as 20% and 80% in the human genome, respectively. Beyond targeted insertions, the system was also shown to catalyze large-scale genomic rearrangements, including inversions up to ∼930 kb and deletions spanning ∼130 kb. Recently, Pelea et al. independently demonstrated ISCro4 activity using both plasmid-based and all-RNA delivery, achieving programmable multi-kilobase excisions and insertions in human cells, highlighting the versatility of bridge recombinase as programmable genome editors capable of megabase-scale chromosome engineering.^50^

However, several challenges remain. Current bridge recombinase systems exhibit lower insertion efficiencies compared to well-optimized tyrosine or large serine recombinase systems. Off-target recombination at unintended genomic loci has been observed and requires further mitigation through continued RNA and protein engineering. Additionally, comprehensive long-term safety profiling in therapeutically relevant primary cell types and *in vivo* models is still needed.

### DNA transposons

Transposases are another type of fourth-generation gene editors and are enzymes that facilitate the movement of DNA segments known as transposons (jumping genes) from one location to another in a genome. The core mechanism involves mobilization of a DNA intermediate through a “cut and paste” mechanism (Figures 2D–2F). Transposases are the key enzymes that catalyze this transposition and maintain stable, efficient integration of genetic material. In general, transposon systems are used because they offer high cargo capacity, reduced immunogenicity, are able to integrate into diverse cell types, and do not rely on a host DSB repair pathway.

### DNA-only transposons

Of all of the DNA-only transposases, the most widely used are the *piggyBac* and *Sleeping Beauty* systems. Both are powerful, non-viral gene delivery systems with stable integration for DNA.^51^ Both systems consist of two components: a transposase enzyme and an inverted terminal repeat (ITR)-flanked DNA cargo. The transposase recognizes the ITRs, cuts the cargo from the donor DNA, and then catalyzes insertion into the host genome at specific target sites TTAA for *piggyBac* or TA for *Sleeping Beauty* (Figure 2D). Unlike SSRs, DNA transposons do not require pre-installed landing sites; however, their integration profile is semi-random as they integrate at TA or TTAA motifs that occur abundantly throughout the human genome.

*Sleeping Beauty* (SB100× and its variant SB200×) integrates transgene of up to 10 kb and is highly efficient compared to its original synthetic ancestor^52^^,^^53^ but its excision leaves behind a small genomic footprint. By contrast, piggyBac offers seamless excision, and also demonstrates higher transposition efficiency,^54^^,^^55^^,^^56^ larger cargo capacity exceeding 100 kb. These properties make piggyBac particularly attractive for stable gene transfer in CAR T cell therapy.^51^^,^^57^

While valuable in stable gene integration, both systems integrate semi-randomly across the genome, raising concerns about insertional mutagenesis.^58^ Sleeping Beauty shows a relatively unbiased genome-wide integration profile, whereas piggyBac exhibits a preference for transcriptional regulatory regions and transcription start sites.^59^ Efforts to direct integration to defined genomic loci through fusion of transposases with programmable DNA-binding domains, such as zinc fingers or TALE arrays, have shown promise but remain limited in efficiency and specificity.^60^^,^^61^ More recently, piggyBac fused to Cas9 has shifted the system from protein-based target recognition to RNA-guided specificity which has been described in the “hybrid integration platforms.”^62^

### CASTs

CASTs are RNA-guided transposition systems that use CRISPR-Cas system to guide DNA insertions without double-strand breaks, which is typical of traditional CRISPR-Cas systems. Unlike classical CRISPR-Cas systems, CASTs leverage guide RNA to direct Tn7-like transposase proteins to a specific genomic locus and thus achieve efficient site-specific DNA integration, making them valuable tools in bacterial genome engineering and potentially in eukaryotic genome engineering.^63^ So far, two major classes, type I-F and type V-K CASTs, have been developed for genome engineering applications.

### Type I-F CAST

Type I-F CASTs mediate RNA-guided DNA integration through the QCascade complex, which consists of Cas6, Cas7, Cas8, and a guide RNA bound to the transposition factor TniQ.^64^ The guide RNA within QCascade serves as the programmable element of the system that directs target recognition through RNA-DNA base pairing, analogous to CRISPR guide RNAs. Upon DNA recognition, QCascade recruits TnsC, which in turn engages the heteromeric TnsA-TnsB transposase that catalyze excision of the donor transposon and precise insertion at the genomic locus specified by the guide RNA (Figure 2E).^64^ In bacteria, they achieve highly specific DNA insertion of up to 10 kb at efficiencies up to 100%, making them an attractive tool for genome engineering.^65^ However, their application in mammalian systems was limited by poor activity (0.1%–1%). Recently, Liu and Stenberg’s group leveraged directed evolution to optimize the CAST machinery and developed evoCAST that achieved 10%–20% integration efficiencies at genomic targets in human cells.^66^ This represents the first demonstration of a CAST system with therapeutically relevant activity in mammalian cells.

The system has been demonstrated to function in mammalian cells and is highly reprogrammable. However, the large size of the multi-component system comprising seven distinct proteins and a guide RNA presents significant challenges for *in vivo* delivery.

### Type V-K CAST

Type V-K CASTs are a more compact class of the CRISPR transposons that, unlike QCascade in type I-F CAST, utilize a single Cas12 k effector protein and a guide RNA as a targeting module.^67^ The Cas12 k and a guide RNA bind to a DNA protospacer and via recruitment of transposition machinery (TnsB, TnsC, and TniQ) catalyzes insertion of the donor DNA (Figure 2F). Typically, this insertion happens 57–67 bp downstream of a protospacer adjacent motif (PAM) sequence.^68^ In prokaryotes, V-K, CASTs are highly efficient for inserting multi-kilobase DNA cargos (up to 80%) but because they lack the TnsA subunit, the cut and paste excision is not clean and hence often yield undesirable fusions of the donor plasmid backbone along with the cargo. This challenge has been addressed by a recent engineering innovation, helix, in which a nicking homing endonuclease was fused to TnsB to mimic the function of TnsA, thereby improving product purity up to 99.4% without compromising its integration efficiency.^33^^,^^68^

Although V-K CASTs are well-suited for large DNA cargo integration in prokaryotes, their activity in mammalian cells is very low. Recently, Liu et al. optimized a compact V-K CAST from uncultivated microbes for nuclear localization and activity in human cells. However, the efficiencies remain low around 1%–2% in HEK293T cells and <0.3% in hematopoietic lines.^33^^,^^68^ Rational engineering or directed evolution approaches are expected to further enhance their activity in mammalian cells for potential therapeutic application.

Although V-K CASTs are more compact than I-F CASTs with four effector protein components compared to seven in I-F systems still face challenges for *in vivo* delivery, and in addition, their clinical application is limited by low efficiency.

### Retrotransposons

Retrotransposons are mobile genetic elements that mobilize through a “copy and paste” mechanism, whereby an RNA intermediate is reverse transcribed into a cDNA copy, which is then integrated elsewhere in the genome (Figures 2G–2I).^69^ Based on the core mechanism used for mobilization, they are further classified into long terminal repeat (LTR) retrotransposons, mobile group II introns, and non-LTR retrotransposons.

### LTR retrotransposons

LTR retrotransposons are characterized by the presence of LTRs flanking the internal coding region that encodes for structural and enzymatic polyproteins Gag and Pol, respectively.^70^ The structural proteins form virus-like particles inside which reverse transcription takes place, and the enzymatic proteins reverse transcriptase and integrase catalyze reverse transcription and integration of cDNA copy into the target genome (Figure 2G). Retroviruses like human immunodeficiency virus (HIV) and murine leukemia virus (MLV) use a process similar to LTR retro-transposition to integrate their genome into the host cell genome.^71^ In addition to classical LTR retrotransposons, retroviruses encode Env structural protein that allows virus particles to infect another cell.

Retroviral vectors are widely used for stable gene transfer in basic and clinical research for generating stable cell lines and *ex vivo* gene therapies, respectively.^72^^,^^73^^,^^74^ However, a major limitation is the lack of precise control over integration sites. HIV (lentiviral) vectors integrate preferentially within active transcription units, whereas MLV vectors favor near transcription start sites and CpG islands.^75^ These preferences enrich integrations in regulatory regions and proto-oncogene loci, which is particularly undesirable for *in vivo* therapeutic gene integration because it increases the risk of insertional mutagenesis and dysregulated host gene expression.^75^^,^^76^

### Mobile group II introns: Targetrons

Targetrons are gene editing tools that have derived from mobile group II introns and are naturally occurring, self-splicing RNA enzymes that can integrate its own cDNA into defined genomic sites via target-primed reverse transcription (TPRT).^77^^,^^78^ These integrases’ intron-encoded reverse transcriptase (IEP) catalyzes intron RNA splicing to form RNA lariat, reverse splicing to insert RNA lariat into a target DNA, cleavage of the target DNA, and reverse transcription to facilitate intron DNA integration into the target genome (Figure 2H). Targetron has become well known among the fourth-generation gene editors because of its precise and efficient gene insertion ability in bacterial and organellar genomes (targeting frequencies from 1% to 100% with selection).^79^ However, their performance in the mammalian genome remains limited, typically <1%, requiring further engineering or evolution to unlock their full potential in mammalian cells.^79^ What’s unique about these integrases is their ability to be dual directional. Specifically, its intron can be targeted to insert in either an antisense or sense orientation relative to target gene transcription by selecting target sequences in opposite DNA strands.^79^ Their targeting specificity is determined by short exon-binding sequences within the intron RNA that base pair directly with complementary target DNA sequence.^80^ These interactions are simple and computationally predictable; therefore, they can be engineered to retarget basically any DNA sequence, making them a valuable tool for targeted DNA insertion. For gene delivery, the IEP open reading frame within the intron RNA is replaced by the cargo and the IEP is expressed in *trans*.^81^ Although the system is highly programmable and can be delivered efficiently as ribonucleoprotein complex (intron RNA and IEP) presents significant challenges for clinical application due to limited efficiency.

### Non-LTR retrotransposons

Unlike LTR retrotransposons that use integrase to integrate a cDNA copy, non-LTR retrotransposons, like mobile group II introns, use TPRT mechanism to complete their mobilization. Unlike group II introns that are found in bacteria and eukaryotic organelles, non-LTR elements make up a significant portion of the eukaryotic genome.^82^ Non-LTR elements such as long- and short-interspersed nuclear elements (LINES and SINES) constitute about one-third (∼1 billion bp) of the human genome and are the only active mobile elements in humans.^83^ However, their integration process is semi-random, preferentially at polyT sites (e.g., 5′-TTTT/AA-3′ for LINE-1), which makes them unsuitable for site-specific gene integrations.^82^^,^^84^

### R2 retrotransposons

R2 retrotransposons are a type of non-LTR retrotransposons that encode their own reverse transcriptase and endonuclease protein for site-specific integration.^79^^,^^85^^,^^86^ R2 retrotransposons naturally integrate their own cDNA into defined sites within highly conserved 28 S rDNA sites via TPRT, the similar RNA to DNA integration strategy used by mobile group II introns (Figure 2I). However, unlike group II introns whose specificity is determined by short exon-binding sequences within the intron RNA, R2 target site is dictated by DNA-binding domains of the R2 protein and partially by homology sequences in the R2 RNA.^87^

Recent structural and biochemical studies revealed that only the 5′ and 3′ untranslated regions (UTRs) of the R2 RNA are required for the retrotransposon-mediated integration, allowing the open reading frame to be replaced with a therapeutic RNA cargo to create all-RNA-mediated gene integration system.^88^ Leveraging this, the zebra finch R2 (R2Tg) was identified as one of the several R2 orthologs active in human cells and was adapted to deliver non-R2 cargo RNAs.^85^^,^^89^^,^^90^ This all-RNA delivery system offers several advantages for *in vivo* use such as transient expression of integration tools, compatibility with existing and efficient mRNA delivery technologies, making the system attractive for future therapeutic integration applications. Nevertheless, efficiency remains modest, highlighting the need for future engineering and evolution.

### Hybrid integration platforms

#### Prime editor-integrase hybrid PASTE/PASSIGE/evoPASSIGE: Cas9+Bxb1

Scientists have been able to combine CRISPR with integrases to develop programmable addition via site-specific targeting elements (PASTE), fixing the issues with DBSs for SSRs.^91^ PASTE utilizes a Cas9 nickase coupled with a reverse transcriptase and a serine integrase Bxb1 to first insert a landing site into the genome, and then integrate a desired DNA payload. The study demonstrated that PASTE is able to integrate sequences up to 36 kb in size with efficiencies equal to or even greater than traditional methods, and importantly, with far fewer off-target events. This makes PASTE a suitable vehicle for gene therapy and synthetic biology, especially in primary human and non-dividing cells where HDR is typically not effective.

A similar prime-editing-assisted site-specific integrase gene editing (PASSIGE) tool based on prime editing and Bxb1 transposition was developed by Anzalone et al.^92^ They demonstrated targeted integration of >10 kb DNA cargos in the mammalian genome with up to 6% efficiency. The tool was further evolved and engineered (eePASSIGE) improving the integration efficiency by 4.2-fold compared to PASSIGE.^93^ Notably, this method outperformed PASTE on average by 16-fold, demonstrating an efficient method for the targeted integration of large DNA cargos in mammalian cells.

### Cas9-transposase fusion TransCRISTI: Cas9+piggyBac

TransCRISTI (transposase-CRISPR-mediated targeted integration) is a type of hybrid fourth-generation gene editing technology that merges the site-specific targeting of CRISPR-Cas9 with excision capability of the piggyBac transposase. Specifically, TransCRISTI takes SpCas9 and combines it with an integration deficient piggyBac double mutant to create a hybrid that has the site targeting of Cas9 sites as well as piggyBac’s donor excision. When these two are combined, it creates an almost “all star” technology that can make homology-independent insertions without donor embedded small guide RNA (sgRNA) sites or plasmid backbone carry-over.^62^

TransCRISTI achieved 3%–4% site-specific integration in HEK293T cells at *AAVS1* and *PML* loci, which is significantly higher than CRISPR-based knock in strategies based on homology-independent repair pathways such as CRISPR HITI.^62^ In selected isogenic cells, TransCRISTI achieved 72% on-target insertion at *AAVS1* loci, demonstrating efficient site-specific integration.

### Cas9-retrotransposase fusion STITCHR: cas9+R2 retrotransposon

The third type of hybrid fourth-generation gene editing technology is a mix between Cas9 systems and R2 retrotransposons or site-specific target-primed insertion through targeted CRISPR homing of retroelements (STITCHR). This hybrid is similar to TransCRISTI as it uses SpCas9 nickase to reprogram R2 retrotransposons to have programmable, scarless gene integration.^94^ The system was discovered in a computer system of thousands of nLTR retrotransposons. Specifically, it was discovered through the identification of R2Tg (zebra finch) and R2Tocc orthologues, which both demonstrated high target primed reverse transcription activity and tolerance to homology reprogramming.^94^ Scientists fused SpCas9H840A to R2ToccΔ1-169 to generate a reprogrammable gene editor that has the ability to install edits from single bases to 12.7 kb payloads without having to make double-strand breaks or donor DNA templates.

For STITCHR, Cas9 introduces a nick that primes R2 reverse transcriptase to copy RNA payloads into the genome with homology arms that direct the specificity. The reprogrammed targeting enabled efficient insertions at AAVS1, NOLC1, LMNB1, EMX1 loci having a 3%–11% integration efficiency and minimal indels.^94^ STITCHR was found to support a diversity of edit types: precise insertions, deletions, gene replacements, multiplexed knock ins, and even functioned in both dividing and non-dividing cells.^94^ Not to mention, unlike prime editing from earlier generations, STITCHR was able to achieve long-range, scarless integration with single-copy precision, which was confirmed by PacBio HiFi sequencing and droplet digital PCR (ddPCR).^94^ STITCHR’s off-target profiling showed high specificity with a lot of the insertions made being on-target sites.

Altogether, STITCHR has expanded on programmable gene editing technologies and takes the best tools from Cas9 and R2 retrotransposons to create a super strong tool moving forward.

### Emerging hybrid technologies using Fanzor

Beyond Cas9-guided hybrid editors such as PASTE/PASSIGE (Cas9 + recombinase), TransCRISTI (Cas9 + transposase) and STITCHR (Cas9 + R2 retrotransposon), there is growing interest in replacing Cas9 with alternative compact RNA-guided effectors like Cas12f^95^^,^^96^ or OMEGA (obligate mobile element guided activity) endonucleases, including TnpB,^97^ IscB,^95^ and Fanzor.^98^ Fanzor is a eukaryotic OMEGA system that uses a compact ωRNA to direct the nuclease to specific DNA sequences and mediate programmable cleavage in mammalian cells.^98^ However, current Fanzor systems are limited by low efficiency and a narrow target-adjacent motif (TAM) requirement.^99^ Recent engineering efforts (enNlovFz2) have improved its cleavage efficiency by 11.1-fold; however, still performs less effectively than Cas9.^100^ Further engineering and research is needed to enhance its performance and expand its targeting capabilities for potential use in integration-based genome editors.

### Delivery considerations for integration-based platforms

Delivery is still one of the biggest challenges for translating fourth-generation gene editors into therapies. While many of the platforms in this review have been validated *in vitro* or *ex vivo*, achieving efficient and safe *in vivo* delivery of large integration systems presents distinct challenges that are different from the issues with earlier generations.

Adeno-associated viral (AAV) vectors have been the preferred vehicle for *in vivo* delivery because of their favorable tissue tropism and the ability to transduce both dividing and non-dividing cells. However, their packaging capacity is restricted to just 4.7 kb, which is incompatible with the size of most fourth-generation integration platforms.^101^^,^^102^ For example, systems in this paper such as type I-F CASTs, PASTE, and PASSIGE all require large effector proteins, guide RNAs, and donor DNA templates that collectively exceed the packaging capacity of single AAV. Dual or triple AAV strategies have been explored to address this challenge but these approaches result in reduced efficiency and increased manufacturing costs.^101^

Lipid nanoparticles (LNPs) are the most clinically advanced non-viral delivery platform that have unrestricted cargo capacity, lower immunogenicity, and the ability to be re-dosed without triggering anti-capsid immune responses.^103^ LNPs are useful for delivering mRNA effectors and guide RNAs at the same time, and therefore are compatible with platforms whose functional components can be delivered entirely as RNA. Bridge recombinase systems, for example, consist of a single recombinase protein and a non-coding bridge RNA, both of which can be encoded as mRNA and bridge RNA respectively, enabling an all-RNA delivery format. Similarly, non-LTR retrotransposon systems such as R2 encode their protein machinery as a single open reading frame and utilize an RNA intermediate for TPRT, making them inherently compatible with mRNA-based LNP delivery. This all-RNA compatibility offers a significant translational advantage over multi-component DNA-dependent systems such as CASTs and PASTE, which requires co-delivery of large effector proteins, guide RNAs, and exogenous donor DNA templates that collectively exceed the practical limits of current LNP formulations.

A promising emerging strategy is the hybrid AAV-LNP approach, where the integration donor DNA template is delivered via AAV while the integration machinery is provided as mRNA via LNP. This approach keeps the donor template stable while maintaining transient expression of the integration machinery, reducing the risk of off-target integrations. This strategy was demonstrated by Zakas et al. for Sleeping Beauty transposase, where an LNP-delivered SB100× mRNA combined with AAV-delivered transposon cargo enabled a stable genomic integration and long-term transgene expression in both mouse and non-human primate liver models.^104^ This combined approach improved gene expression 10-fold over conventional AAV alone while requiring 5-fold fewer vectors.

Engineered viral-like particles (eVLPs) have also become a rapidly advancing delivery platform. Unlike AAVs, eVLP’s are able to deliver their cargo as ribonucleoproteins, thereby substantially reducing the risk of prolonged off-target editing and unwanted cargo integration. Optimized eVLPs have been developed that are cable of delivering base editor ribonucleoproteins (BE-eVLPs) and prime editor ribonucleoproteins (PE-eVLPs) at therapeutically acceptable efficiencies across multiple different tissues.^105^^,^^106^ Their modular envelope glycoprotein architecture permits tissue-specific targeting through envelope engineering, a feature that could be leveraged to direct integration-based editors to specific tissues *in vivo*.^107^

The choice of delivery platform will ultimately depend on the size and complexity of each integration system. Smaller systems with RNA-guided components may be more readily compatible with LNP and eVLP delivery, whereas larger multi-component platforms such as CASTs and PASTE will require further delivery innovations before clinical translation can be realized.

### Conclusion and future perspectives

Integration-based editing allows the generation of large, precise genetic insertions, which could be readily applied to many established disease-causing loci. Unfortunately, well-characterized safety profiles, on-target efficiency and specificity, as well as optimal delivery modalities, remain as significant barriers to their widespread application.

Herein, we have introduced and characterized the broad groups of currently available gene integrating systems. SSRs have been shown to yield high efficiency for targeted or controlled gene rearrangements, while viral integrases are still highly effective for stable gene delivery. Newer transposon systems offer a non-viral alternative with high cargo capacity; CASTs such as type I-F and type V-K and other RNA-guided integrases have allowed for programmable, DSB-free DNA insertion with high directionality but low to moderate efficiency; and the discovery of Fanzor and OMEGA systems have offered promise to the field moving forward.

Despite the marked progress in the engineering of these fourth-generation gene editors, their direct application as an *in vivo* therapy will require substantial work. The effector proteins of many of the fourth-generation systems are large, exceeding the ∼4.7 kb packaging capacity of AAV vectors.^101^ However, effector protein size alone does not preclude clinical translation as demonstrated by the use of mRNA-LNP platforms to deliver large-sized CRISPR gene editing tools in patients. The primary challenge for fourth-generation tools is not the size of effector proteins but rather multi-component architecture of these systems, requiring coordinated delivery and expression of these components within the same cell. This multi-component requirement increases the complexity of formulation, manufacturing, and dosing, which serves as a major barrier to clinical translation. Additionally, ongoing research will be needed to fully characterize the off-target activity profile of gene integration tools. This is technically challenging particularly for the integrase systems that exhibit some degree of promiscuity in their integration site preference. Such promiscuity increases the complexity of off-target sites mapping, as potential insertion sites are distributed across the genome rather than confined to predictable loci. Long read whole genome sequencing using platforms such as PacBio high-fidelity or Oxford Nanopore, remains the most comprehensive strategy currently available for unbiased detection of off-target integrations.^108^ However, long read sequencing is limited by lower throughput, greater cost, and higher per-base error rate compared to short read platforms, thereby affecting routine safety profiling in translational applications.^108^^,^^109^ The move to integrase-based editing has revolutionized the prospects of genomic medicines, and this technology is well positioned to offer real therapies for a variety of monogenic and complex conditions. The ongoing refinement and optimization of these tools will unlock a new paradigm for definitive genetic correction.

## Acknowledgments

A.W.H. is supported by the Australian National Health and Medical Research Council Fellowship.

## Author contributions

All authors contributed equally to this manuscript.

## Declaration of interests

The authors declare no competing interests.

## Declaration of generative AI and AI-assisted technologies in the writing process

During the preparation of this work, the authors used Claude to improve the scientific tone of the manuscript. After using this tool, the authors reviewed and edited the content as needed and take full responsibility for the content of the published article.
